# Assessing the Sensitivity and the Clinical Impact of the 2023 American College of Rheumatology/EULAR Classification Criteria in Obstetric Antiphospholid Syndrome: Findings From a Multicenter Italian Cohort With a Long‐Term Follow‐Up

**DOI:** 10.1002/acr.80040

**Published:** 2026-04-29

**Authors:** Francesca Ruffilli, Cecilia Nalli, Francesca Crisafulli, Carlo Garaffoni, Marcello Govoni, Giulia Voltarel, Alba C. Pozzi, Beatrice Maranini, Ettore Silvagni, Alessandra Bortoluzzi, Angela Tincani

**Affiliations:** ^1^ Department of Medical Sciences, University of Ferrara, Italy; Rheumatology Unit Azienda Ospedaliero‐Universitaria S.Anna, Cona Ferrara Italy; ^2^ Rheumatology and Clinical Immunology Unit – ERN ReCONNET ASST Spedali Civili Brescia Italy; ^3^ Department of Clinical and Experimental Sciences University of Brescia Brescia Italy

## Abstract

**Objective:**

The aim of this study was to evaluate the sensitivity of the 2023 American College of Rheumatology (ACR)/EULAR classification criteria for antiphospholipid syndrome (APS) in a real‐world cohort of women diagnosed with primary obstetric APS (oAPS) and to assess their ability to identify patients at risk of future pregnancy complications.

**Methods:**

We conducted a multicenter retrospective study on women diagnosed with primary oAPS by expert rheumatologists between 2006 and 2023 in two Italian rheumatology centers. All patients were retrospectively classified using both the 2006 Sydney and the 2023 ACR/EULAR classification criteria. Follow‐up at one and five years after clinical diagnosis was analyzed to identify new obstetric events and assess associations with baseline classification status.

**Results:**

Our cohort included 122 women. At baseline, 75.4% of patients fulfilled the Sydney criteria and 23% met the 2023 ACR/EULAR criteria. The main reasons for exclusion under the 2023 ACR/EULAR criteria were insufficient clinical score due to isolated fetal losses (56.4%), insufficient laboratory score due to isolated IgM antiphospholipid antibody positivity (4.3%), or a combination of both (5.3%). During five years of follow‐up, more than one‐quarter of the patients who became pregnant experienced new obstetric events. The majority of these patients did not fulfill the 2023 ACR/EULAR criteria.

**Conclusion:**

The 2023 ACR/EULAR classification criteria demonstrate low sensitivity (23%) in women with primary oAPS and fail to identify those at risk of future events. These findings underscore the need for cautious clinical judgment in managing oAPS: misuse of these criteria may result in undertreatment and increase the risk of recurrent obstetric complications.

## INTRODUCTION

Antiphospholipid syndrome (APS) is an autoimmune prothrombotic disorder defined by the presence of antiphospholipid antibodies (aPLs), and characterized by a range of clinical manifestations, most notably vascular thrombosis and obstetric complications. Obstetric APS (oAPS) is a distinct subset with both thrombotic and nonthrombotic pathogenic mechanisms in which pregnancy morbidity is the predominant manifestation, encompassing both fetal events—such as recurrent early miscarriages and/or unexplained fetal losses—and maternal complications, including early‐onset preeclampsia and/or placental insufficiency, which may lead to preterm birth.^1^

**SIGNIFICANCE & INNOVATIONS**

- This study reveals the low sensitivity of the 2023 American College of Rheumatology/EULAR criteria in patients with obstetric antiphospholipid syndrome (oAPS), largely due to the minimal weighting of fetal losses and isolated IgM antiphospholipid antibody positivity.
- The new criteria fail to identify women at risk of future pregnancy complications.
- To conduct clinical studies in oAPS, patients should be stratified according to their clinical features rather than classified according to formal criteria.
- These findings highlight the critical role of clinical judgment and individualized management in patients with oAPS.

The first classification criteria for APS were introduced in 1999 and subsequently revised in 2006 as the Sydney criteria.^2^, ^3^ Because there are no diagnostic criteria for APS, these classification criteria have been widely used in clinical practice to support diagnosis and guide management. In 2023, the American College of Rheumatology (ACR) and EULAR published new classification criteria for APS (2023 ACR/EULAR classification criteria), representing a methodologically rigorous update to improve specificity and enable more accurate identification of homogeneous patient cohorts, thereby facilitating research progress in APS.^4^ The updated criteria introduced a stratification system based on aPL profiles and expanded the clinical domain to include features such as microvascular involvement, thrombocytopenia, and cardiac manifestations. Additionally, the definition of obstetric complications has been revised, with different weights assigned to fetal losses, whether early or late, and to placental complications (preeclampsia and/or placental insufficiency with severe features, before 34 gestational weeks [GWs]).

Since their release, the new criteria have sparked extensive debate. There is general consensus that they offer high specificity (99%) but relatively low sensitivity (84%),^4^ a limitation particularly evident in the obstetric domain.^5^, ^6^, ^7^, ^8^ Notably, the sensitivity of the 2023 ACR/EULAR classification criteria in patients whose primary disease manifestations are obstetric complications remains poorly investigated. Moreover, most of the validation studies conducted thus far include patients with APS secondary to other autoimmune diseases, complicating the interpretation of certain newly included clinical features. We aimed to investigate the long‐term outcomes of women diagnosed with primary oAPS, focusing on the occurrence of new obstetric events over a five‐year follow‐up. In this context, we assessed the sensitivity of the 2023 ACR/EULAR classification criteria and evaluated their ability to identify patients at risk for new obstetric events in a real‐world clinical setting during medium‐ to long‐term follow‐up.

## MATERIALS AND METHODS

### Study design

This multicenter, observational, retrospective study involved women with primary oAPS. The research cohort included patients observed at the Rheumatology and Clinical Immunology Unit of Aziende Socio Sanitarie Territoriali Spedali Civili in Brescia (Italy) and the Rheumatology Unit of Sant'Anna Hospital in Ferrara (Italy), encompassing both outpatient and inpatient settings. Eligible patients were those diagnosed with primary oAPS based on clinical judgment of rheumatologists with expertise in APS, between January 2006 and December 2023. Because validated diagnostic criteria for APS are currently unavailable, the clinical diagnosis was established through a comprehensive evaluation of compatible obstetric manifestations and positive aPL titers, following the exclusion of other maternal or fetal causes for pregnancy complications. Patients with secondary APS, including those with a concomitant diagnosis of systemic lupus erythematosus (SLE) or other defined systemic autoimmune rheumatic diseases, were excluded.

This study investigated the clinical outcomes of a multicenter primary oAPS cohort over a five‐year period. We evaluated the sensitivity of the 2023 ACR/EULAR classification criteria compared to the 2006 Sydney criteria in existing cohorts of patients with primary oAPS, defined after the publication of the 2006 Sydney criteria. Furthermore, we assessed the performance of the 2023 criteria in identifying patients at risk for new obstetric events, analyzing how these tools reflect clinical risk at both one and five years after diagnosis. The study was conducted in full accordance with the principles outlined in the Declaration of Helsinki. Ethical approval was obtained from the Ethics Committee of Azienda Ospedaliera‐Universitaria in Cona (approval code 1/2025/Oss/AOUFe).

### Data collection

Baseline data included demographic and medical history information such as anthropometric data, ethnicity, smoking status, exposure to hormonal therapy, inherited thrombophilia, comorbidities, organ‐specific autoimmune disorders (autoimmune thyroiditis, celiac disease, vitiligo, inflammatory bowel disease, multiple sclerosis), age at diagnosis, clinical manifestations of APS, laboratory findings, and treatments. Systemic autoimmune diseases were excluded by design. Ethnicity was obtained from medical records.

Each patient was classified according to both the 2006 Sydney and the 2023 ACR/EULAR classification criteria for APS. A full description of both sets of criteria is provided in Supplementary Tables S1 and S2. According to the 2006 criteria, patients were classified as having APS if at least one clinical and one laboratory criterion were met. For the 2023 ACR/EULAR criteria, classification required meeting the “entry criteria”—defined as at least one documented clinical criterion plus a positive aPL test within three years—and achieving a weighted score of at least three points from the clinical domains (domains 1–6) and three points from the laboratory domains (domains 7 and 8). Antibody thresholds were determined via standardized enzyme‐linked immunosorbent assay, with medium‐high titers for anticardiolipin antibody (aCL) and anti–β2–glycoprotein I (aβ2GPI) IgG/IgM categorized as either moderate (40–79 units) or high (>80 units). To account for the discrepancy between clinical practice and research standards, patients with a confirmed clinical diagnosis who failed to meet the required score for the 2023 ACR/EULAR classification criteria were categorized as having noncriteria oAPS.

Follow‐up data were collected at one‐ and five‐year time points after diagnosis and included changes in autoantibody profiles, the occurrence of new APS‐related events, new uncomplicated pregnancies, treatment modifications, and identification of additional patients fulfilling the 2023 ACR/EULAR classification criteria. Cumulative pregnancy incidence at five years was defined as the total number of women experiencing at least one pregnancy during the entire follow‐up interval (zero to five years). Follow‐up was based on data extracted from medical charts of patients who were regularly evaluated approximately every 6 to 12 months, or earlier in the case of new pregnancies, clinical complications, or significant changes in their clinical status. All data were anonymized and managed using the REDCap system.^9^

A risk profile stratification was established based on the following characteristics: high‐risk aPL profile is characterized by persistent lupus anticoagulant (LAC) positivity on at least two occasions spaced 12 weeks apart, double positivity (any combination of LAC, aCL, or aβ2GPI), triple positivity (all three antibodies), or persistently high antibody titer^10^; medium‐risk aPL profile includes medium‐to‐high titer of aCL (IgG/IgM) and/or aβ2GPI (IgG/IgM) above 40 GPL/MPL units but with negative LAC; finally, low‐risk profile involves isolated aCL or aβ2GPI antibodies at low to medium titer, especially if transiently positive.^11^ Regarding data integrity, missing values were identified for some clinical and laboratory variables due to the retrospective nature of the study. These have been explicitly reported in the results and within each table by specifying the actual denominator for each variable.

### Statistical analysis

Given that this sensitivity validation study included exclusively women with a clinically confirmed diagnosis of oAPS, only sensitivity could be estimated for the 2023 ACR/EULAR and 2006 Sydney classification criteria. The lack of a reference population without disease precluded the estimation of specificity, false‐positive rates, and receiver operating characteristic curves. Statistical analyses were conducted by reporting the mean values (±SD) or median values (interquartile range [IQR]) for normally distributed and nonnormally distributed variables, respectively, whereas categorical variables were expressed as numbers and percentages of patients. Continuous variables were compared between patients fulfilling or not fulfilling the 2023 ACR/EULAR criteria (noncriteria oAPS), as well as between those with and without new clinical events at one‐ and five‐year follow‐ups. The latter analysis was restricted to patients who achieved pregnancy during the follow‐up period. Student's *t*‐test was used for normally distributed variables, whereas Wilcoxon test was used for nonnormally distributed variables. For comparing categorical variables, Fisher test and the chi‐square test were used. Statistical significance was defined by a *P* value <0.05. Odds ratios (ORs) with 95% confidence intervals (CIs) were calculated to estimate the risk of obstetric events between patients who met the ACR/EULAR classification criteria and those who did not meet it (noncriteria oAPS).

## RESULTS

### Cohort description and 2023 ACR/EULAR classification criteria assessment

We enrolled 122 women diagnosed with primary oAPS with a mean age (±SD) at diagnosis of 35 (±7) years. The main demographic and anamnestic data of the cohort, as well as clinical and laboratory features, are reported in Table 1. At diagnosis, 102 patients (83.6%) received antiplatelet therapy—47.5% had newly initiated treatment, whereas 36.1% continued therapy started before diagnosis. For these patients, antiplatelet therapy was often started before a formal oAPS diagnosis to manage recurrent pregnancy complications or other coexisting risk factors. Prophylactic heparin was used by 36 patients (29.5%) during pregnancy. Anticoagulation was rare (four patients [3.2%]: two taking warfarin and two taking direct oral anticoagulants). Glucocorticoids and hydroxychloroquine were used by 4.1% and 6.6% of patients, respectively. No conventional synthetic disease‐modifying antirheumatic drugs (DMARDs) or biologic DMARDs were administered. A total of 13 patients (10.7%) received no treatment at diagnosis because they were not seeking pregnancy and presented with an aPL profile that did not require prophylaxis outside of pregnancy.

**Table 1:** Demographic and anamnestic data of the cohort at baseline (N = 122)*

Characteristics	n/total n (%)^a^
White	103/122 (84)
BMI, mean (±SD), kg/m^2^	24.7 (±5)
Current smokers	11/110 (10)
Previous exposure to hormone therapy	45/88 (51.1)
Inherited thrombophilia^b^	44/86 (51.2)
Comorbidities	
Autoimmune thyroiditis	17/122 (13.9)
Arterial hypertension	16/122 (13.1)
Diabetes mellitus	6/122 (4.9)
Dyslipidemia	4/122 (3.3)
Neoplasm	3/122 (2.5)
Chronic renal failure	1/122 (0.8)
Age at diagnosis, mean (±SD), y	35 (±7)
Obstetric history	
Miscarriage at ≤10 GWs^c^	70/122 (57.4)
Fetal death at >10 GWs^c^	57/122 (46.7)
Uncomplicated pregnancy^c^	43/122 (35.2)
Complicated pregnancy^c^	27/122 (22)
Pregnancies complicated by severe PE^c^	18/122 (14.8)
Pregnancies complicated by eclampsia^c^	1/122 (0.8)
Pregnancies complicated by PI^c^	15/122 (12.3)
Preterm delivery^c^	22/122 (18)
Perinatal deaths (within 28 days of life)	8/122 (11)
Thrombotic events	
Venous thrombosis^c^	9/122 (7.4)
Arterial thrombosis^c^	1/122 (0.8)
Laboratory tests	
Low platelets in patient's history (≤130,000 × 10^9^/L)	9/122 (7.4)
High‐risk aPL profile	31/122 (25.4)
Medium‐risk aPL profile	70/122 (57.4)
Low‐risk aPL profile	21/122 (17.2)
ANA positivity	45/107 (42.1)
Low levels of C3 and/or C4	9/99 (9.1)

* ANA, antinuclear antibody; aPL, antiphospholipid antibody; BMI, body mass index; GW, gestational week; PE, preeclampsia; PI, placental insufficiency.

^a^ Percentages are calculated based on available data for each variable; missing values were excluded from the denominators.

^b^ Inherited thrombophilia included antithrombin III, protein C, and protein S deficiency; elevated homocysteine; and factor II, factor V, and MTHFR (C677T) mutation (heterozygous or homozygous).

^c^ There was at least one event per patient.

According to the 2006 Sydney criteria, 92 patients (75.4%) were classifiable as having APS. Based on the 2023 ACR/EULAR criteria, 95 of 122 patients (77.9%) met the “entry criteria”; however, only 28 patients (23%) fulfilled the new classification criteria by scoring at least three points in both the clinical and laboratory domains. A total of 64 women who had previously been classified as having APS according to the 2006 Sydney criteria did not reach the required composite score under the new classification criteria. In our cohort, the sensitivity of the 2006 Sydney criteria was 75.4%, whereas the 2023 ACR/EULAR criteria demonstrated a markedly lower sensitivity of 23%.

Figure 1 illustrates the reasons for exclusion from the 2023 ACR/EULAR classification criteria. For 54 patients (57.5%), the minimum clinical score of 3 was not achieved, primarily due to recurrent early pregnancy losses and/or early fetal deaths (32 patients), late fetal deaths (21 patients), or a single case of arterial thrombosis in the context of a high cardiovascular risk profile, all occurring without additional clinical manifestations. For four patients (4.3%), the laboratory domain score was insufficient because they tested positive only for IgM aPL (aCL and/or aβ2GPI) at moderate to high titers. For the remaining nine patients (9.6%), neither the clinical nor the laboratory score thresholds were met. These included early pregnancy losses associated with fluctuating LAC positivity combined with IgM aPL (two patients), early pregnancy losses with isolated IgM aPL positivity (four patients), and late fetal losses associated with either combined fluctuating LAC and IgM aPL positivity (one patient) or isolated IgM aPL positivity (two patients). Patients who met the 2023 ACR/EULAR classification criteria were significantly younger at diagnosis (mean ± SD 33 ± 4 years vs 36 ± 7 years, *P* = 0.014) and showed a higher prevalence of antinuclear antibody (ANA) positivity (73.9% vs 33.3%, *P* < 0.001) and high‐risk aPL profile (57.1% vs 16%, *P* < 0.001) compared to patients who were not classifiable (noncriteria oAPS).

**Figure 1:**
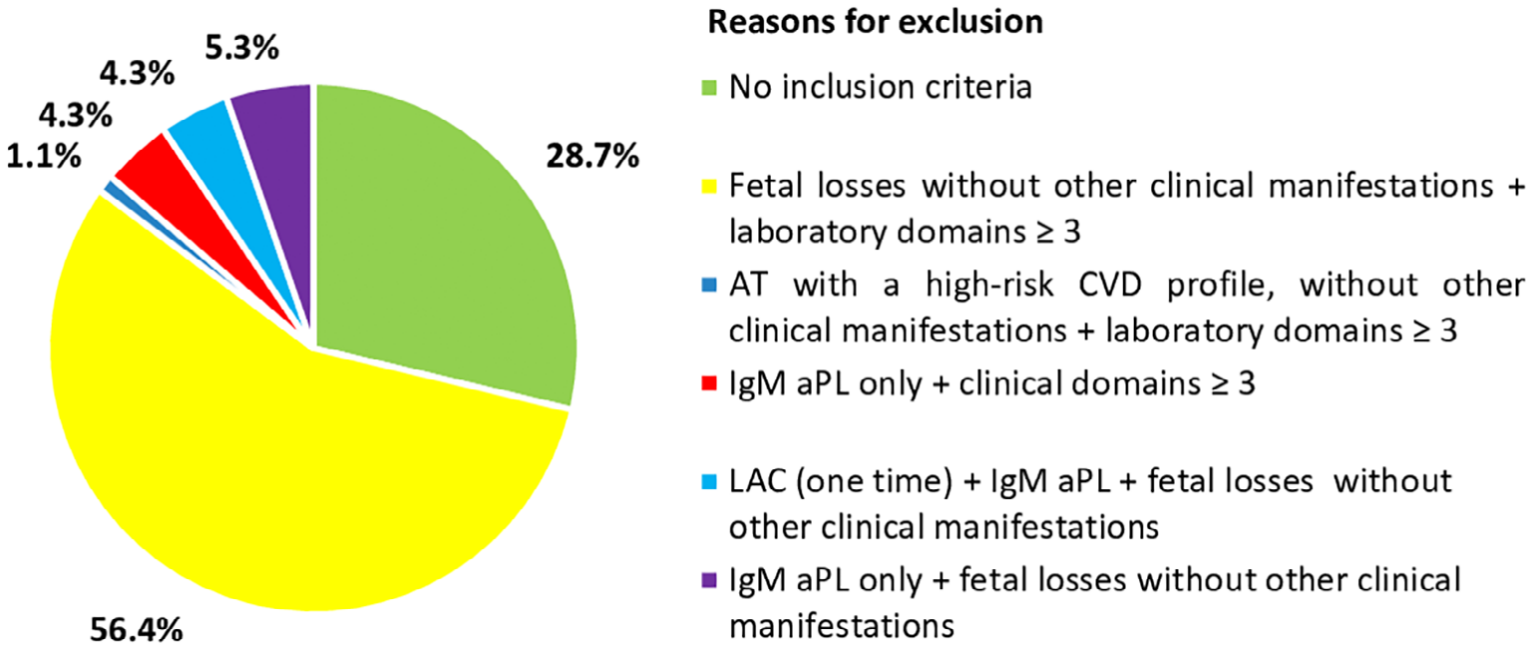
Reasons for exclusion from classification based on 2023 American College of Rheumatology/EULAR classification criteria. aPL, antiphospholipid antibody; AT, arterial thrombosis; CVD, cardiovascular disease; LAC, lupus anticoagulant.

Table 2 shows the distribution of the eight domains of the new classification criteria, divided between patients who met the criteria and those who did not. Among the clinical domains, the most significant difference was observed in the obstetric domain (domain 4): in patients who fulfilled the criteria, fetal losses were less represented, whereas placental disorders were more common. Furthermore, significant differences emerged in the macrovascular (domain 1) and hematology (domain 6) domains, which further contributed to the classification of patients according to the new criteria. Other significant differences were observed in the laboratory domains (domains 7 and 8): patients who met the criteria had a higher prevalence of persistent LAC positivity and high‐titer IgG aPL positivity. In contrast, LAC positivity in a single finding, medium‐titer IgG aPL positivity, and medium‐/high‐titer IgM aPL positivity were more frequent among those who did not meet the criteria. The increased serological burden in patients who were positive for the criteria was confirmed by significantly higher domain 7 (median [IQR] 5 [0–5] vs 0 [0–1], *P* = 0.006) and combined domain 7 and 8 scores (median [IQR] 5 [4–10] vs 4 [4–5], *P* < 0.001) compared to the group with noncriteria oAPS.

**Table 2:** Distribution of the eight domains (domains 1–8) of the 2023 ACR/EULAR classification criteria in patients who fulfilled the inclusion criteria (N = 95) and comparison between patients who did (N = 28) and did not fulfill (N = 67) the classification criteria*

Domains	Total (N = 95),^a^ n (%)	Score ≥ 6 (N = 28),^a^ n (%)	Score < 6 (N = 67),^a^ n (%)	*P* value
Clinical domains				
Domain 1: macrovascular (VTE)				**<0.001**
VTE with a high‐risk VTE profile	1/95 (1.1)	1/28 (3.6)	0/67	
VTE without a high‐risk VTE profile	7/95 (7.4)	6/28(21.4)	1/67 (1.5)	
Domain 2: macrovascular (AT)				>0.9
AT with a high‐risk CVD profile	1/95 (1.1)	0/28	1/67 (1.5)	
AT without a high‐risk CVD profile	0/95	0/28	0/67	
Domain 3: microvascular				0.085
Suspected	2/95 (2.1)	2/28 (7.1)	0/67	
Established	1/95 (1.1)	0/28	1/67 (1.5)	
Domain 4: obstetric				**<0.001**
At least three consecutive prefetal deaths (<10 GWs) and/or early fetal deaths (10–15 + 6 GWs)	41/95 (43.2)	1/28 (3.6)	40/67 (59.7)	
Fetal death (16–33 + 6 GWs) without PE/PI with severe features	29/95 (30.5)	5/28 (17.9)	24/67 (35.8)	
PE with severe features (<34 GWs) or PI with severe features (<34 GWs)	13/95 (14)	13/28 (46.4)	0/67	
PE with severe features (<34 GWs) and PI with severe features (<34 GWs)	8/95 (8.4)	6/28 (21.4)	2/67 (3)	
Domain 5: cardiac valve				0.085
Thickening	2/95 (2.1)	2/28 (7.1)	0/67	
Vegetation	0/95	0/28	0/67	
Domain 6: hematology				**0.002**
Thrombocytopenia (PLTs 20–130 × 10^9^/L)	5/95 (5.3)	5/28 (17.9)	0/67	
Laboratory domains				
Domain 7: LAC positivity				**0.003**
One time	6/95 (6.3)	0/28	6/67 (9)	
Persistent	31/95(33)	16/28(57.1)	15/67(22.4)	
Domain 8: aPL test by ELISA				**0.001**
IgM aCL and/or aβ2GPI positivity (moderate–high)	17/95 (18)	1/28 (3.6)	16/67 (23.9)	
IgG aCL and/or aβ2GPI positivity (moderate)	40/95 (42)	9/28 (32.1)	31/67 (46.3)	
IgG aCL or aβ2GPI positivity (high)	17/95 (18)	5/28 (17.9)	12/67 (17.9)	
IgG aCL and aβ2GPI positivity (high)	9/95 (9.5)	7/28 (25)	2/67 (3)	

*Note:* Bold values indicate statistical significance at the *p* < 0.05 level.

* aβ2GPI, anti–β2–glycoprotein I; aCL, anticardiolipin antibody; ACR, American College of Rheumatology; aPL, antiphospholipid antibody; AT, arterial thrombosis; CVD, cardiovascular disease; ELISA, enzyme‐linked immunosorbent assay; GW, gestational week; LAC, lupus anticoagulant; PE, preeclampsia; PI, placental insufficiency; PLT, platelet; VTE, venous thromboembolism.

^a^ Percentages are calculated based on available data for each variable; missing values were excluded from the denominators.

### Follow‐up and pregnancy outcomes

Data at the one‐year follow‐up were available for 95 of the 122 enrolled patients because 27 (22.1%) were lost to follow‐up due to discontinuation of clinical monitoring at our centers. During this period, 42 of these women (44.2%) experienced at least one new pregnancy: 28 (66.7%) had uncomplicated pregnancies, whereas 14 (33.3%) developed new obstetric complications. New obstetric complications included six patients with one episode of spontaneous miscarriage before the 10th GW, one patient with two early miscarriages, three patients with fetal losses after the 10th GW, and four patients with placental disorders (preeclampsia or eclampsia). At the five‐year follow‐up, 61 patients remained under observation. Among them, a cumulative total of 40 women (65.6%) had experienced one or more pregnancies during the total follow‐up period. Within this group, 28 women (70%) had uncomplicated outcomes, whereas 12 (30%) experienced at least one new obstetric complication. These events included six early miscarriages, one fetal loss after the 10th GW, one patient with both an early and a late pregnancy loss, and four patients with preeclampsia or eclampsia. No major thrombotic events were reported during the study period.

Tables 3 and 4 summarize the baseline demographic and clinical characteristics of patients who became pregnant during each follow‐up period. Patients who experienced complicated pregnancies were significantly more likely to be ANA positive (71.4% vs 17.9%, *P* = 0.001 at one year; 75% vs 25%, *P* = 0.005 at five years) and to exhibit triple aPL positivity (28.6% vs 0%, *P* = 0.009 at one year of follow‐up), compared to those with uncomplicated outcomes. Thromboprophylaxis was more frequently administered among patients with uncomplicated pregnancies (96.4% vs 78.6% at one year; 92.9% vs 91.7% at five years), although these differences did not reach statistical significance.

**Table 3:** Baseline characteristics of patients with at least one new pregnancy within one year of diagnosis (N = 42) and comparison between patients with uncomplicated pregnancies (N = 28) and those with new obstetric events (N = 14)*

Variables	Total (N = 42),^a^ n (%)	No events (N = 28),^a^ n (%)	New events (N = 14),^a^ n (%)	*P* value
BMI, mean (±SD), kg/m^2^	25.9 (±5.5)	26.2 (±5.8)	25.3 (±5.1)	0.7
Current smokers	5/42 (11.9)	5/28 (17.9)	0/14	0.2
Autoimmune thyroiditis	6/42 (14.3)	2/28 (7.1)	4/14 (28.6)	0.2
Inherited thrombophilia	12/31 (38.7)	10/21 (47.6)	2/10 (20.0)	0.4
ANA positivity	15/42 (35.7)	5/28 (17.9)	10/14 (71.4)	**0.001**
C3, mean (±SD), mg/dL	126 (±31)	125 (±29)	126 (±35)	>0.9
C4, mean (±SD), mg/dL	24 (±8)	26 (±9)	22 (±7)	0.11
Triple aPL positivity^b^	4/42 (9.5)	0/28	4/14 (28.6)	**0.009**
aPL profile				0.3
Low risk	10/42 (23.8)	8/28 (28.6)	2/14 (14.3)	
Medium risk	23/42 (54.8)	16/28 (57.1)	7/14 (50)	
High risk	9/42 (21.4)	4/28 (14.3)	5/14 (35.7)	
Thromboprophylaxis				0.1
Yes	38/42 (90.5)	27/28 (96.4)	11/14 (78.6)	
No	4/42 (9.5)	1/28 (3.6)	3/14 (21.4)	
LDASA	11/42 (26.2)	7/28 (25)	4/14 (28.6)	>0.9
LDASA and heparin	27/42 (64.3)	20/28 (71.4)	7/14 (50)	0.19
2006 Sydney criteria				0.3
Yes	31/42 (73.8)	19/28 (67.9)	12/14 (85.7)	
No	11/42 (26.2)	9/28 (32.1)	2/14 (14.3)	
2023 ACR/EULAR criteria				0.2
Yes	7/42 (16.7)	3/28 (10.7)	4/14 (28.6)	
No	35/42 (83.3)	25/28 (89.3)	10/14 (71.4)	

*Note:* Bold values indicate statistical significance at the *p* < 0.05 level.

* ACR, American College of Rheumatology; ANA, antinuclear antibody; aPL, antiphospholipid antibody; BMI, body mass index; LDASA, low‐dose aspirin.

^a^ Percentages are calculated based on available data for each variable; missing values were excluded from the denominators.

^b^ Positivity was of lupus anticoagulant with anticardiolipin antibody (IgG and/or IgM) and anti–β2–glycoprotein I (IgG and/or IgM).

**Table 4:** Baseline characteristics of patients with at least one new pregnancy within five years of diagnosis (N = 40) and comparison between patients with uncomplicated pregnancies (N = 28) and those with new obstetric events (N = 12)*

Variables	Total (N = 40),^a^ n (%)	No events (N = 28),^a^ n (%)	New events (N = 12),^a^ n (%)	*P* value
BMI, mean (±SD), kg/m^2^	23.5 (±4.1)	24.1 (±4.4)	21.8 (±2.8)	0.2
Current smokers	6/40 (15)	5/28 (17.9)	1/12 (8.3)	0.6
Autoimmune thyroiditis	7/40 (17.5)	3/28 (10.7)	4/12 (33.3)	0.2
Inherited thrombophilia	16/30 (53.3)	12/22 (54.4)	4/8 (50.0)	0.8
ANA positivity	16/40 (40)	7/28 (25)	9/12 (75)	**0.005**
C3, mean (±SD), mg/dL	123 (±30)	125 (±28)	118 (±35)	0.6
C4, mean (±SD), mg/dL	25 (±8)	26 (±8)	23 (±8)	0.3
Triple aPL positivity^b^	4/40 (10)	3/28 (10.7)	1/12 (8.3)	>0.9
aPL profile				0.9
Low risk	8/40 (20)	5/28 (17.9)	3/12 (25)	
Medium risk	23/40 (57.5)	16/28 (57.1)	7/12 (58.3)	
High risk	9/40 (22.5)	7/28 (25)	2/12 (16.7)	
Thromboprophylaxis				>0.9
Yes	37/40 (92.5)	26/28 (92.9)	11/12 (91.7)	
No	3/40 (7.5)	2/28 (7.1)	1/12 (8.3)	
LDASA	9/40 (22.5)	6/28 (21.4)	3/12 (25)	>0.9
LDASA and heparin	28/40 (70)	20/28 (71.4)	8/12 (66.7)	>0.9
2006 Sydney criteria				>0.9
Yes	31/40 (77.5)	22/28 (78.6)	9/12 (75)	
No	9/40 (22.5)	6/28 (21.4)	3/12 (25)	
2023 ACR/EULAR criteria				0.7
Yes	9/40 (22.5)	7/28 (25)	2/12 (16.7)	
No	31/40 (77.5)	21/28 (75)	10/12 (83.3)	

*Note:* Bold values indicate statistical significance at the *p* < 0.05 level.

* ACR, American College of Rheumatology; ANA, antinuclear antibody; aPL, antiphospholipid antibody; BMI, body mass index; LDASA, low‐dose aspirin.

^a^ Percentages are calculated based on available data for each variable; missing values were excluded from the denominators.

^b^ Positivity was of lupus anticoagulant with anticardiolipin antibody (IgG and/or IgM) and anti–β2–glycoprotein I (IgG and/or IgM).

In the first year after diagnosis, recurrences of pregnancy complications were observed in 14 patients; of these, 10 (71.4%) did not fulfill the 2023 ACR/EULAR classification criteria at baseline, compared to only 2 patients (14.3%) who failed to meet the 2006 Sydney criteria (these 2 patients did not fulfill either set of criteria). Likewise, 10 of 12 patients (83.3%) with recurrences within five years failed to meet the 2023 classification criteria, whereas 3 patients (25.0%) did not satisfy the 2006 Sydney criteria. As illustrated in Figure 2, the 2006 Sydney criteria identified a greater proportion of patients who subsequently developed new obstetric events compared to the 2023 ACR/EULAR criteria. The latter failed to adequately recognize patients at risk for new obstetric complications (OR 3.33, 95% CI 0.63–17.65 at one year; OR 0.6, 95% CI 0.11–3.43 at five years).

**Figure 2:**
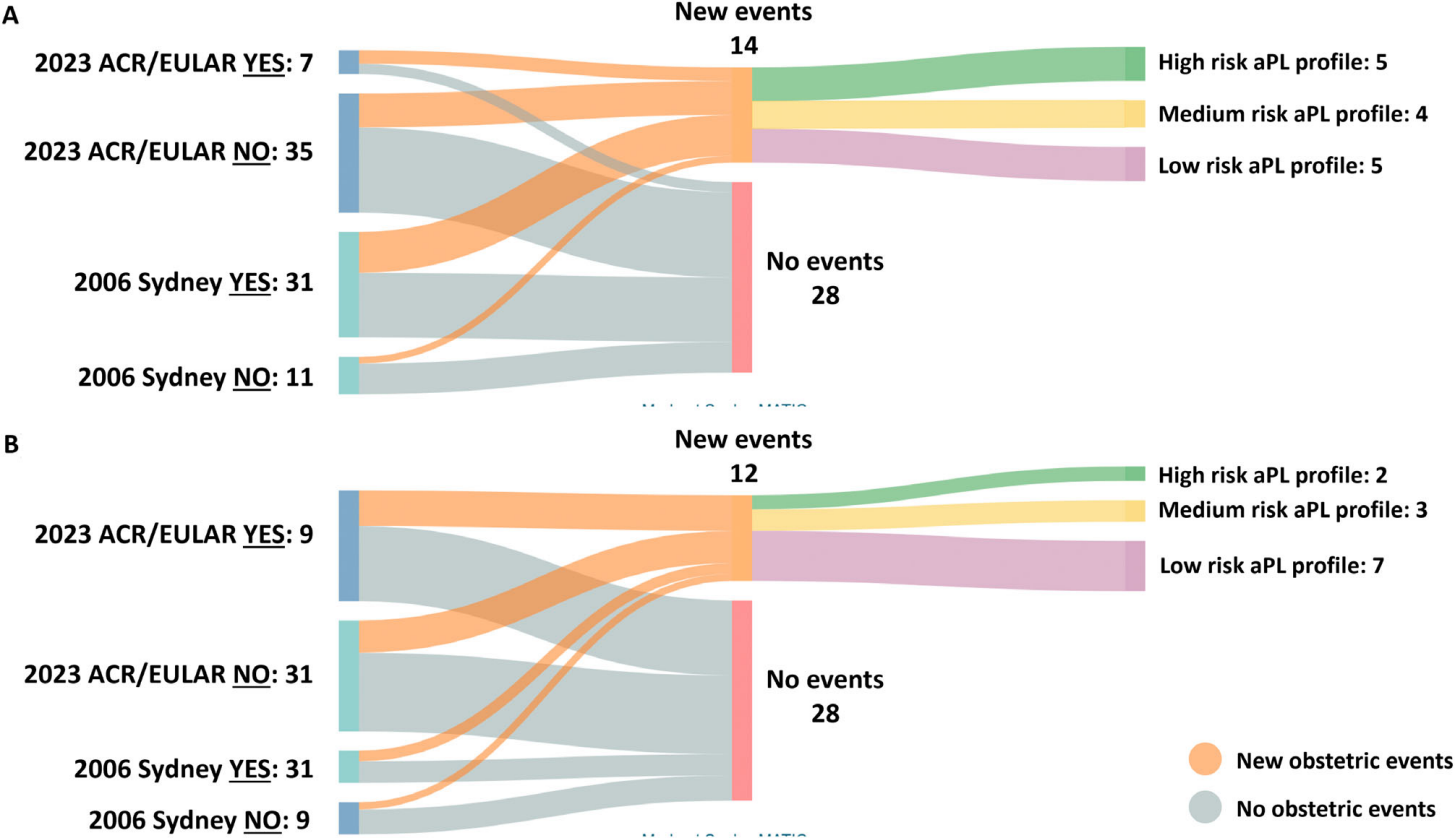
Comparison between the 2023 American College of Rheumatology (ACR)/EULAR and the 2006 Sydney classification criteria for antiphospholipid syndrome in patients who developed new obstetric events within (A) one year and (B) five years of follow‐up, along with the representation of antiphospholipid antibody (aPL) profiles.

When considering the aPL risk profiles at baseline among patients who experienced obstetric events within the first year of follow‐up, five patients (35.7%) had a low‐risk profile, four (28.6%) a medium‐risk profile, and five (35.7%) a high‐risk profile. In this period, one patient exhibited a change in her aPL risk profile, shifting from medium to low risk. Patients classified as low risk all presented at least one additional risk factor for adverse pregnancy outcomes. These included advanced maternal age (≥40 years, n = 2), obesity (body mass index ≥ 30, n = 1), coexisting organ‐specific autoimmune disorders (celiac disease, n = 1; autoimmune thyroiditis, n = 1), ANA positivity (n = 5), and reduced levels of C3 and/or C4 (n = 2).

Similarly, among patients who experienced new obstetric events within five years of follow‐up, seven (58.3%) had a low‐risk aPL profile, three (25%) had a medium‐risk profile, and two (16.7%) had a high‐risk profile at baseline. Only one patient showed a change in his antibody profile over time, shifting from a medium‐ to a low‐risk profile. Among the eight patients with a low‐risk aPL profile, seven had at least one additional risk factor for the development of obstetric complications. Specifically, one patient was aged ≥40 years at diagnosis, two had congenital thrombophilia (MTHFR mutation), two had an organ‐specific autoimmune disease (one with celiac disease and one with autoimmune thyroiditis), six were ANA positive, and one patient showed low complement levels.

## DISCUSSION

Our study provides a comprehensive longitudinal evaluation of a multicenter primary cohort with oAPS, assessing the sensitivity of the 2023 ACR/EULAR classification criteria compared to the 2006 Sydney criteria. Furthermore, it evaluates the ability of these criteria to identify patients at risk of new obstetric complications. The core finding of our work is the significant discrepancy between the 2023 ACR/EULAR classification thresholds and the clinical reality of recurrent obstetric events observed during follow‐up.

The proportion of patients classified as having APS was significantly lower with the 2023 ACR/EULAR criteria than the 2006 Sydney criteria, reflecting a substantial reduction in sensitivity for detecting APS in patients with isolated obstetric manifestations—75.4% with the 2006 Sydney criteria versus only 23% with the new classification system. These observations align with existing evidence showing that the impaired sensitivity of the new criteria is primarily driven by the obstetric domain.^5^, ^6^, ^7^, ^8^, ^12^, ^13^ Notably, although studies in Asian populations have reported a lower sensitivity of the 2023 ACR/EULAR criteria, this trend is even more striking in cohorts of White patients like ours.^12^, ^13^ Furthermore, in our study, a nonnegligible proportion of patients experienced new obstetric events after diagnosis. The most frequently observed events were early miscarriages, followed by placental disorders and fetal losses. Notably, ANA positivity and triple aPL positivity emerged as significant risk factors for adverse pregnancy outcomes, consistent with recent findings by Martínez‐Taboada et al.^12^ It is important to emphasize that patients with a diagnosis of SLE or other systemic autoimmune rheumatic diseases were excluded from our study. Therefore, the high prevalence of ANA positivity observed in women with poor pregnancy outcomes suggests that ANA may serve as an independent immunologic marker of risk within the population with oAPS.^14^ The 2023 ACR/EULAR criteria failed to identify patients who developed new obstetric complications: at both one and five years of follow‐up, the majority of such events occurred in noncriteria oAPS. In contrast, the 2006 Sydney criteria proved more effective in capturing individuals who were at risk. Moreover, although aPL profiles remained largely stable over time, several patients with low‐risk profiles still experienced complications. This aligns with existing evidence suggesting that even low aPL titers may carry clinical relevance, particularly if associated with other potential risk factors for pregnancy.^15^, ^16^ These findings point to the potential role of additional factors acting as “second hits” in the pathogenesis of these events and underline the need for an individualized, comprehensive risk assessment that extends beyond rigid classification frameworks.

The reduced sensitivity of the 2023 ACR/EULAR criteria, particularly evident in the obstetric subset of APS, is largely due to the stricter definitions applied within the obstetric domain. Many patients presenting with three or more consecutive early miscarriages and/or fetal losses, but without additional clinical manifestations, receive only one point in the clinical domain, which is insufficient for classification. Furthermore, changes in the laboratory criteria contribute to the low sensitivity of the new criteria because isolated IgM isotype positivity at moderate–high titer is no longer sufficient for classification. Although these revisions improve specificity, they also exclude clinically relevant cases commonly seen in oAPS.^17^, ^18^ These limitations have significant implications for both research and clinical practice. By restricting classification to severe placental disorders, which occur in only 10% to 15% of patients with APS, the 2023 criteria significantly narrow their applicability, excluding the less severe but more frequent disease phenotypes. Moreover, the scoring system assigns only the highest score within each domain, without accounting for cumulative effects of multiple events, thus it may not adequately capture the clinical complexity of oAPS, in which different obstetric events may stem from distinct pathogenic pathways. Several authors have suggested refinements to better represent the clinical spectrum of oAPS, such as adopting cumulative scoring within domains to account for the additive burden of different obstetric complications (eg, recurrent miscarriage and late fetal loss).^19^ In the laboratory domain, the weighted scoring system based on antibody profile and titer may disadvantage patients with isolated positivity of IgM aCL/aβ2GPI, which, although considered less specific, has nonetheless been associated with adverse pregnancy outcomes.^20^, ^21^

Importantly, our longitudinal follow‐up data demonstrate that a substantial proportion of patients continue to experience new obstetric events even after diagnosis. In this context, it is clinically relevant to note a trend in which patients who experienced events in the follow‐up appeared to receive less intensive treatment. Although this finding did not reach formal statistical significance, it suggests that a gap in early treatment intensification might contribute to adverse outcomes in higher‐risk subsets. This further raises concerns about the adequacy of current classification systems in capturing the full spectrum of individuals who were at risk: the 2006 Sydney criteria identified a considerable number of patients who went on to develop new adverse events during follow‐up, whereas the recently introduced 2023 ACR/EULAR criteria demonstrated a markedly lower capability in detecting such patients. However, beyond the issue of differing sensitivity between classification systems, our data also reinforce the critical role of expert clinical judgment in identifying individuals who were at risk. Notably, several patients who did not fulfill any classification criteria but who were nonetheless clinical diagnosed by experienced physicians developed obstetric complications during follow‐up. Therefore, although classification criteria are valuable for standardization and research, they should not replace individualized clinical evaluation, which remains essential for comprehensive and effective patient care.

Moreover, the application of the new criteria may inadvertently lead to difficulties in accessing therapies.^22^, ^23^ Hence, it is crucial to emphasize that classification criteria are not diagnostic tools and should not be used to guide therapy decisions or deny appropriate care to patients who do not meet strict criteria but have clinical profiles warranting intervention.^24^, ^25^, ^26^ A recent study by Schreiber et al analyzed the impact of the 2023 ACR/EULAR criteria for APS on diagnosis, research, and real‐world practice, with a particular focus on pregnancy‐related aspects of the syndrome.^27^ The study emphasizes the need to ensure that these criteria do not negatively affect patient care and reiterates that they are not meant to serve as diagnostic criteria. Treatment decisions must remain clinical and individualized based on a comprehensive risk profile, including obstetric history, comorbidities, and other contributing factors.

Our study has some limitations. First, the lack of a control group prevents assessment of the specificity and overall accuracy of the new criteria within our cohort. Clinical diagnosis, although reflective of real‐life practice, may introduce variability and potential overdiagnosis in the absence of standardized diagnostic criteria for oAPS. Furthermore, the retrospective study design introduces potential biases related to data quality and collection methods, including the presence of missing data. A further limitation lies in the potential misalignment between our objectives and the original scope of the 2023 ACR/EULAR criteria, which were primarily designed for research classification rather than clinical prognosis. Although applying these criteria in a real‐world setting departs from their intended purpose, this mismatch underscores a critical gap in current strategies for identifying patients at risk for recurrent obstetric events. Nonetheless, our study has significant strengths: it focuses exclusively on primary oAPS, minimizing confounding from systemic autoimmune diseases, and is one of the few studies based on clinical diagnosis, offering a real‐world evaluation of the 2023 ACR/EULAR criteria in clinical practice. Additionally, it incorporates well‐defined follow‐up intervals, enhancing the consistency of data collection. The true strength of our study lies in the postdiagnosis follow‐up, which specifically evaluates the impact of these criteria on the recurrence of obstetric complications.

In conclusion, the 2023 ACR/EULAR classification criteria exhibit low sensitivity in patients with primary oAPS, particularly those presenting with isolated fetal losses and isolated IgM‐predominant aPL profiles. Of note, several obstetric complications were observed during follow‐up in women presenting with a mild oAPS phenotype, particularly among noncriteria oAPS. These findings further highlight a critical limitation of the new criteria, which failed to effectively identify patients at risk for subsequent obstetric events. By excluding many women with milder clinical presentations, who nonetheless experienced clinically relevant complications during follow‐up, the 2023 criteria demonstrate limited utility in real‐world experience. Reliance on 2023 APS classification criteria as diagnostic tools, as sometimes physicians might be tempted to, risks underrecognition and undertreatment of clinically significant cases, potentially exposing patients to preventable adverse outcomes.

## AUTHOR CONTRIBUTIONS

All authors contributed to at least one of the following manuscript preparation roles: conceptualization AND/OR methodology, software, investigation, formal analysis, data curation, visualization, and validation AND drafting or reviewing/editing the final draft. As corresponding author, Dr Ruffilli confirms that all authors have provided the final approval of the version to be published, and takes responsibility for the affirmations regarding article submission (eg, not under consideration by another journal), the integrity of the data presented, and the statements regarding compliance with institutional review board/Declaration of Helsinki requirements. All authors contributed to the investigation, formal analysis of the data, and writing, reviewing, and editing of the manuscript.

## Supporting information


**Disclosure Form**:

**Table S1.** The 2006 Sydney classification criteria for APS.
**Table S2.** The 2023 ACR/EULAR classification criteria for APS.
