# Supplementary material for: Assessing the Sensitivity and the Clinical Impact of the 2023 American College of Rheumatology/EULAR Classification Criteria in Obstetric Antiphospholid Syndrome: Findings From a Multicenter Italian Cohort With a Long‐Term Follow‐Up
Source: Arthritis Care Res (Hoboken). 2026 Apr 29;78(11):1691–700. doi: 10.1002/acr.80040 (PMC13710784; doi:10.1002/acr.80040)
Supplement: Supplementary file 2 [file ACR-78-1691-s002.docx]

Table S1. The 2006 Sydney classification criteria for APS.

*Clinical domains*

1. Vascular thrombosis: one or more clinical episodes of arterial, venous, or small vessel thrombosis, in any tissue or organ.

Diagnosis of thrombosis requires objective confirmation via validated imaging or histopathology. Histopathologic evidence must demonstrate thrombosis without significant inflammation of the vessel wall.

2. Pregnancy morbidity (at least one of the following):

a) One or more unexplained deaths of a morphologically normal fetus at ≥ 10 GW, with normal fetal morphology documented by ultrasound or direct examination;

b) One or more premature births of a morphologically normal neonate at < 34 GW due to: (I) E/severe PE, or (II) recognized features of PI;

c) Three or more unexplained consecutive spontaneous abortions at < 10 GW, after excluding maternal anatomic or hormonal abnormalities and paternal/maternal chromosomal causes.

*Laboratory criteria*

1. LAC positivity on two or more occasions, at least 12 weeks apart, detected according to the guidelines of the International Society on Thrombosis and Haemostasis (Scientific Subcommittee on LAs/phospholipid-dependent antibodies).

2. aCL antibody positivity (IgG and/or IgM isotype) in medium or high titer (i.e. >40 GPL or MPL, or >the 99th percentile), on two or more occasions, at least 12 weeks apart, measured by a standardized ELISA.

3. aβ2GPI antibody positivity (IgG and/or IgM isotype) in titer >the 99th percentile, present on two or more occasions, at least 12 weeks apart, measured by a standardized ELISA, according to recommended procedures.

*Abbreviations*: aβ2GPI: anti-β2glycoprotein I; aCL: anticardiolipin; E: eclampsia; GW: gestational week; LAC: lupus anticoagulant; PE: preeclampsia; PI: placental insufficiency.

| Table S2. The 2023 ACR/EULAR classification criteria for APS. | |
| --- | --- |
| *Domains (D)* | ***Points*** |
| *Clinical domains*  Domain 1: Macrovascular (Venous Thromboembolism [VTE]) |  |
| - VTE with a high-risk VTE profile - VTE without a high-risk VTE profile | **1**  **3** |
| Domain 2: Macrovascular (Arterial Thrombosis [AT]) |  |
| - AT with a high-risk CVD profile - AT without a high-risk CVD profile | **2**  **4** |
| Domain 3: Microvascular |  |
| - Suspected:   One or more of the following: livedo racemosa (exam); livedoid vasculopathy lesions (exam); acute/chronic aPL-nephropathy (exam or lab); pulmonary hemorrhage (symptoms and imaging).   - Established:   One or more of the following: livedoid vasculopathy (pathology); acute/chronic aPL-nephropathy (pathology); pulmonary hemorrhage (BAL or pathology); myocardial disease (imaging or pathology); adrenal hemorrhage (imaging or pathology). | **2**  **5** |
| Domain 4: Obstetric |  |
| - ≥ 3 consecutive pre-fetal (<10 GW) and/or early fetal (10 - 15+6 GW) deaths - Fetal death (16 – 33+6 GW) in the absence of PE/PI with severe features - PE with severe features (<34 GW) or PI with severe features (<34 GW) with/without fetal death - PE with severe features (<34 GW) and PI with severe features (<34 GW) with/without fetal death | **1**  **1**  **3**  **4** |
| Domain 5: Cardiac valve |  |
| - Thickening - Vegetation | **2**  **4** |
| Domain 6: Hematology |  |
| - Thrombocytopenia (PLT 20-130 x10^9^/L) | **2** |
| *Laboratory domains* |  |
| Domain 7: aPL test by coagulation-based functional assay (LAC) |  |
| - Positive LAC (single - one time) - Positive LAC (persistent*) | **1**  **5** |
| Domain 8: aPL test by solid phase assay (aCL and/or aβ2GPI antibody in ELISA [persistent*]) |  |
| - IgM aCL and/or aβ2GPI positivity (moderate-high titer) - IgG aCL and/or aβ2GPI positivity (moderate titer) - IgG aCL or aβ2GPI positivity (high titer) - IgG aCL and aβ2GPI positivity (high titer) | **1**  **4**  **5**  **7** |
| * Persistent: two consecutive positive tests, measured ≥ 12 weeks apart.  *Abbreviations*: aβ2GPI: anti-β2glycoprotein I; aCL: anticardiolipin; BAL: bronchoalveolar lavage; CVD: cardiovascular disease; D: domain; Exam: physical examination; GW: gestational week; Lab: laboratory tests; LAC: lupus anticoagulant; PE: preeclampsia; PI: placental insufficiency; PLT: platelets. | |
